# Efficacy of albumin use in decompensated cirrhosis and real‐world adoption in Australia

**DOI:** 10.1002/jgh3.70029

**Published:** 2024-09-18

**Authors:** Eric Kalo, Scott Read, Asma Baig, Kate Marshall, Wai‐See Ma, Helen Crowther, Cameron Gofton, Kate D Lynch, Siddharth Sood, Jacinta Holmes, John Lubel, Alan Wigg, Geoff McCaughan, Stuart K Roberts, Paolo Caraceni, Golo Ahlenstiel, Avik Majumdar

**Affiliations:** ^1^ Blacktown Clinical School and Research Centre, School of Medicine Western Sydney University Blacktown New South Wales Australia; ^2^ Blacktown Hospital, Western Sydney Local Health District Blacktown New South Wales Australia; ^3^ Storr Liver Centre, The Westmead Institute for Medical Research University of Sydney Westmead New South Wales Australia; ^4^ Royal Prince Alfred Hospital Sydney New South Wales Australia; ^5^ Department of Gastroenterology and Hepatology Royal North Shore Hospital St Leonards New South Wales Australia; ^6^ Department of Gastroenterology and Hepatology Royal Adelaide Hospital, Central Adelaide Local Health Network Adelaide South Australia Australia; ^7^ Faculty of Health and Medical Sciences University of Adelaide Adelaide South Australia Australia; ^8^ Department of Gastroenterology Northern Health Melbourne Victoria Australia; ^9^ Department of Medicine The University of Melbourne Parkville Victoria Australia; ^10^ Department of Gastroenterology St Vincent's Hospital Fitzroy Victoria Australia; ^11^ Department of Gastroenterology Alfred Health Melbourne Victoria Australia; ^12^ Central Clinical School Monash University Melbourne Victoria Australia; ^13^ Hepatology and Liver Transplant Medicine Unit Southern Adelaide Local Health Network Adelaide South Australia Australia; ^14^ Flinders University of South Australia Adelaide South Australia Australia; ^15^ A.W. Morrow Gastroenterology and Liver Centre Centenary Research Institute for Cancer Research and Cell Biology Camperdown New South Wales Australia; ^16^ Australian National Liver Transplant Unit Royal Prince Alfred Hospital Sydney New South Wales Australia; ^17^ Faculty of Medicine and Health University of Sydney Sydney New South Wales Australia; ^18^ Unit of Semeiotics, Liver and Alcohol‐Related Diseases IRCCS Azienda‐Ospedaliera Universitaria di Bologna, EMR Bologna Italy; ^19^ Department of Medical and Surgical Sciences University of Bologna, EMR Bologna Italy; ^20^ Victorian Liver transplant Unit Austin Health Heidelberg Victoria Australia

**Keywords:** albumin, challenges, cirrhosis, complications, decompensation, evidence, infusions, long‐term

## Abstract

The current treatment approach to patients with liver cirrhosis relies on the individual management of complications. Consequently, there is an unmet need for an overall therapeutic strategy for primary and secondary prevention of complications. The clinical potential of long‐term albumin infusions supported by recent clinical trials has expanded its indications and holds promise to transform the management and secondary prevention of cirrhosis‐related complications. This renewed interest in albumin comes with inherent controversies, compounding challenges and pressing need for rigorous evaluation of its clinical potential to capitalize on its therapeutic breakthroughs. Australia is among a few countries worldwide to adopt outpatient human albumin infusion. Here, we summarize currently available evidence of the potential benefits of human albumin for the management of multiple liver cirrhosis‐related complications and discuss key challenges for wide application of long‐term albumin administration strategy in Australian clinical practice. Australian Gastroenterological week (AGW), organised by the Gastroenterological Society of Australia (GESA), was held between 9‐11 September 2022. A panel of hepatologists, advanced liver nurses and one haematologist, were invited to a roundtable meeting to discuss the use of long‐term albumin infusions for liver cirrhosis. management in Australia. In this review, we summarise the proceedings of this meeting in context of the current literature.

## Introduction

The natural history of cirrhosis is a continuum from asymptomatic compensated disease to decompensation and chronic liver failure marked by overt clinical signs. Decompensation represents not only an important landmark in the natural history of cirrhosis but a prognostic watershed, resulting in high short‐term mortality.^1^

**Figure 1:**
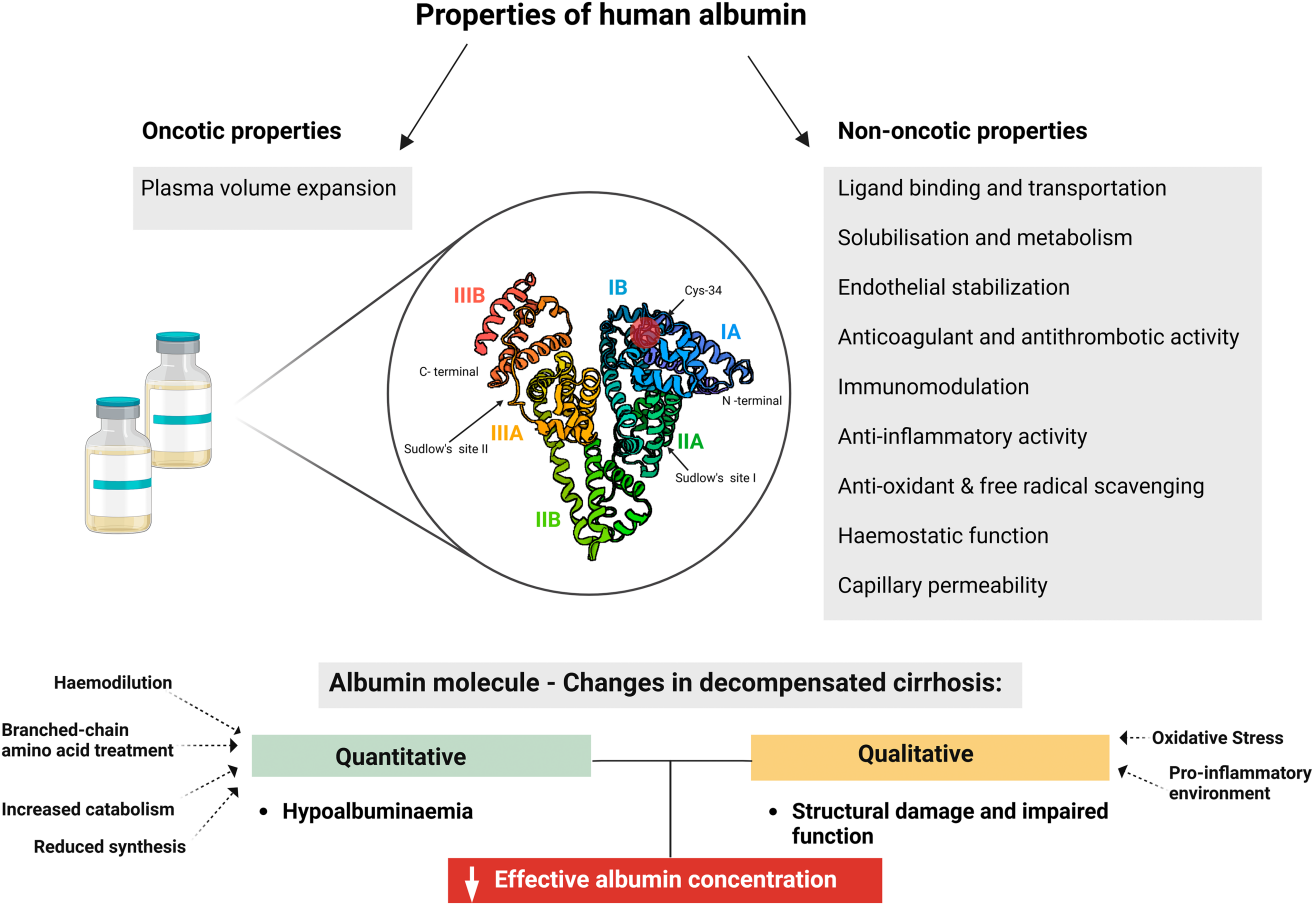
Properties of human albumin and the main changes occurring in patients with decompensated cirrhosis.

Albumin plays an important role in the management of decompensated cirrhosis in Australia, reflecting global trends in hepatology. In patients with decompensated cirrhosis, there is not only a reduction in total serum albumin concentration due to a decrease in hepatic synthetic function, hemodilution, and increased catabolism, but there also exists significant structural and functional alterations of the human albumin (HA) molecule itself (Fig. 1). These changes modify the nononcotic properties of HA, such as antioxidant, anti‐inflammatory, immunomodulatory, and endothelium stabilization properties.^2^, ^3^ Oxidative damage of Cys‐34 residue represents the most frequent qualitative alteration that can occur to the albumin molecule.^4^, ^5^ This is relevant in cirrhosis and portal hypertension as Cys‐34 residue is involved in scavenging the free radical nitric oxide and reactive oxygen species. In addition, the serum albumin molecule may undergo significant dimerization and truncation that can amplify its dysfunction.^6^, ^7^ These qualitative alterations of the albumin molecule progressively accumulate with post‐transcriptional changes, resulting in a decrease in the “effective albumin concentration (eAlb)” and have been correlated with an increase in cirrhosis severity and mortality and carries a greater prognostic power.^8^, ^9^ It is an independent predictor, better than total albumin concentration (tAlb) of future adverse events, such as the short‐term ACLF development (within 30 days) or medium‐term mortality (within 90 days). eAlb can be estimated from the relative amount of native albumin (nAlb), which is the relative abundance of the native albumin isoform, presenting fully preserved structure quantified by LC–MS analysis and the tAlb measured by routine lab methods [eAlb = nAlb (%) × tAlb (g/dL)/100].^9^ Notably, even a subtle reduction in serum albumin has been reported to be a predictor for both, clinical decompensation and death among patients with compensated cirrhosis.^10^

**Figure 2:**
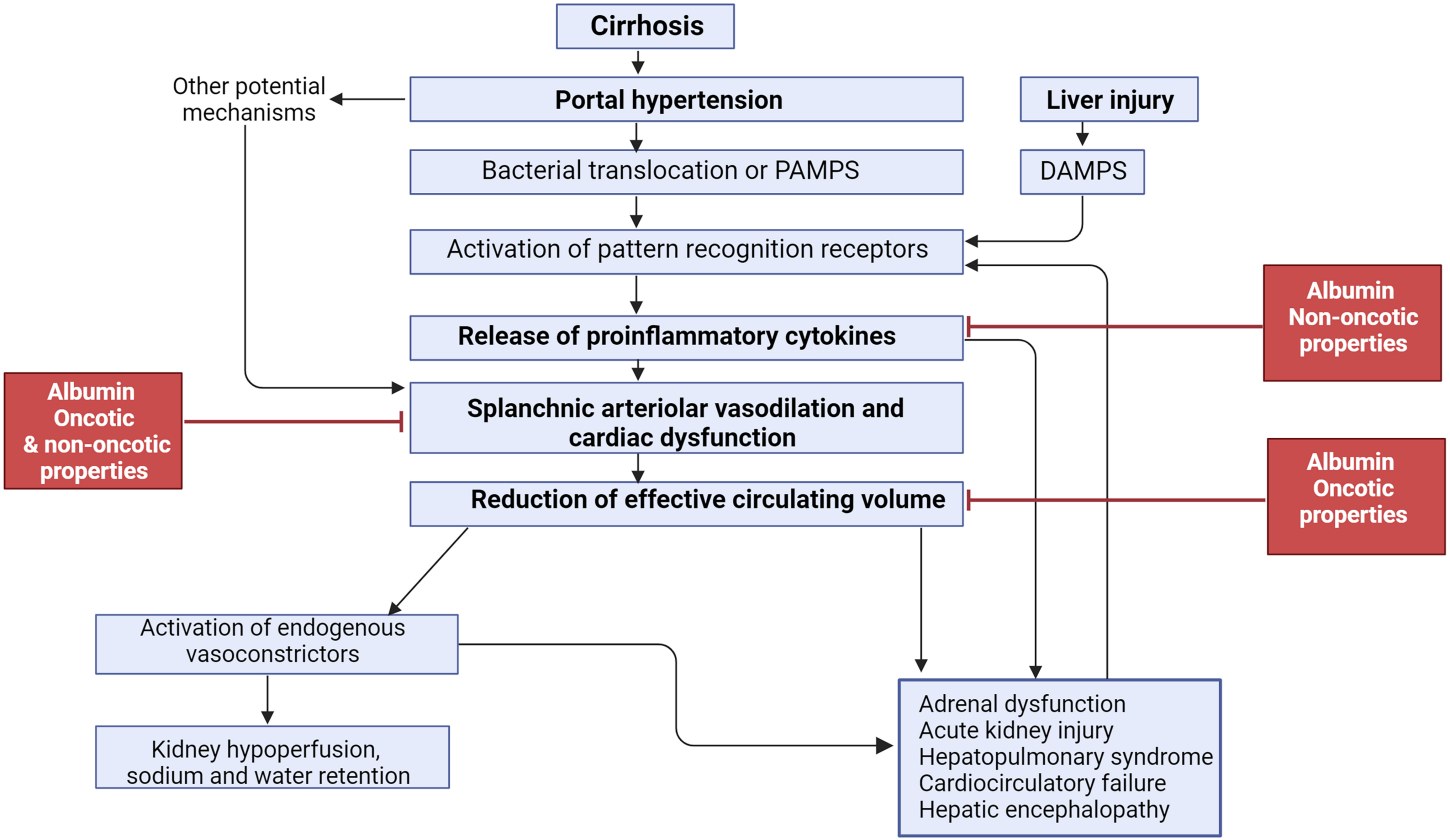
Pathophysiological basis of albumin use in decompensated cirrhosis.

Many complications of cirrhosis (e.g., ascites, renal failure) have been largely attributed to effective hypovolemia resulting from peripheral arterial vasodilation. Persistent inflammation, oxidative stress, and circulatory and immune dysfunction, which remain unopposed by ineffective systemic albumin concentrations, contributes to the development of end‐stage liver disease.^11^, ^12^ Consequently, HA should theoretically act as a potential multitarget agent to counteract effective hypovolemia and attenuate key drivers of decompensation (Fig. 2).

Beyond volume expansion, the potential benefits and proven efficacy of exogenous HA infusions for the management of selected liver cirrhosis related complications is well established for large‐volume paracentesis (LVP) (>5 L), acute kidney injury (AKI), and hepatorenal syndrome (HRS).^13^, ^14^, ^15^ Further to these, the indications for HA are expanding due to emerging literature describing the long‐term administration of HA in ambulatory decompensated patients,^16^, ^17^ acute decompensation,^18^ intrinsic or post‐renal AKI, post‐liver transplantation, nonspontaneous bacterial peritonitis (SBP) infections, hyponatraemia, and hepatic encephalopathy (HE)^19^ (see Box 1).

Box 1 **Human albumin in chronic liver disease: Indications for short‐term and long‐term administration**

**Table:** 

Accepted indications for short‐term human albumin (HA) administration: Spontaneous bacterial peritonitis (SBP) Large‐volume paracentesis Acute kidney injury (AKI) > stage 1A Combined with terlipressin for hepatorenal syndrome (HRS)‐AKI Controversial indications for short‐term HA administration: Septic shock Hyponatremia Overt hepatic encephalopathy (HE) in patients with liver cirrhosis and hypoalbuminemia Non‐SBP infection Long‐term HA can be considered in specific settings for: Uncomplicated ascites requiring diuretic therapy

## Long term use of HA for ascites

The long‐term use of HA in patients with decompensated liver cirrhosis represents a turning point in cirrhosis management, as the aim is to modify the natural history of disease, that is, to reduce hospitalizations and improve survival. Three randomized, placebo‐controlled trials (RCTs) and one nonrandomized pilot study have recently investigated the long‐term use of HA in patients with cirrhosis and ascites^16^, ^17^, ^20^:

In 2018, the ANSWER study (The human Albumin for the treatmeNt of aScites in patients With hEpatic ciRrhosis) was conducted as a multicenter, randomized, pragmatic, open‐label trial.^16^ This study aimed to investigate the impact of HA on 431 patients with cirrhosis and uncomplicated ascites, that is, in the absence of infection or HRS. Patients were randomly allocated to one of two groups: those receiving standard medical treatment (SMT) (*n* = 213) and those receiving SMT in conjunction with HA (*n* = 218). The HA regimen consisted of an initial loading dose of 40 g administered twice weekly for the first 2 weeks, followed by a maintenance dose of 40 g weekly thereafter. The primary endpoint of the study was the mortality rate at 18 months, evaluated based on the difference in events and survival time analysis in patients included in the modified intention‐to‐treat and per‐protocol populations. Notably, the group receiving SMT plus HA demonstrated a significant improvement in survival at 18 months (Kaplan–Meier estimates showed 77% survival in the SMT plus HA group *versus* 66% in the SMT group; *P* = 0.0285). Additionally, there was a reduced requirement for LVP (HR = 0.48, 62% *vs* 34%, *P* < 0.0001), and fewer events of decompensation and complications related to cirrhosis were observed.

The results of the ANSWER study were confirmed in a subsequent prospective, nonrandomized trial, commonly known as the “refractory ascites trial,” of 70 patients with cirrhosis and refractory ascites^21^: 45 patients were nonrandomly assigned to receive SMT plus long‐term administration of HA at the doses of 20 g twice weekly *versus* 25 patients receiving only SMT. The cumulative incidence of 24‐month mortality was significantly lower in patients treated with HA than in patients receiving SMT alone (41.6% *vs* 65.5%; *P* = 0.032). On multivariate analysis, besides age and MELD, HA was the only other independent factor protective against death and was also associated with a lower cumulative incidence of hospitalization with hepatic decompensation.

The MACHT (Midodrine and Albumin for Cirrhotic patients in the waiting list for liver Transplantation) study, a multicenter, randomized, double blinded placebo‐controlled trial, also explored the effects of long‐term infusion of HA (40 g every 15 days) in 196 patients, of whom 99 received HA, with cirrhosis and ascites while waitlisted for liver transplantation.^20^ Unfortunately, the MACHT trial did not meet its primary endpoint of combined liver‐related complications (HE, bleeding, HRS, SBP, hyponatremia) at 12 months.

This divergence between the ANSWER and MACHT trials outcomes may be explained by differences in study design (Table 1): First, the duration of HA administration in the ANSWER study was longer than 1 year (with median length of 14.5 months) *versus* only 63 days in the MACHT study, suggesting a potential time‐dependent effect of HA infusions. Second, the authors of the MACHT study detected suppression of the activity of the renin‐angiotensin‐aldosterone systems (RAAS), suggesting that clinically beneficial effects on circulatory function may require a longer duration or higher dose of HA. Third, the dose of HA was twofold higher in the ANSWER study, resulting in a rise of serum HA from 31 g/L at baseline to approximately 40 g/L within 1–2 months. Conversely, serum HA has increased from 30 to 35 in the HA arm and from 30 to 39 in the placebo group, respectively, in the MACHT study. Of note, no significant change was observed in HA arm as compared with placebo. Fourth, patients recruited to the ANSWER study had lower Model of end‐stage liver disease (MELD) scores compared with the MACHT trial (12–13 *vs* 17–18), suggesting that more advanced hepatic disease may have led to increased post‐transcriptional modification of the infused HA, rendering its clinically relevant non‐oncotic properties less effective. Finally, inherent bias could have been introduced in the control arm of ANSWER study who did not receive fluid or weekly medical supervision. A post hoc analysis of the ANSWER trial demonstrated that baseline serum HA should not guide the decision to start HA therapy and that the serum HA target threshold to be pursued is 40 g/L to achieve desired benefits.^22^ In fact, this analysis demonstrated that on‐treatment serum HA concentration at 1 month predicted the probability of 18‐month overall survival, which was greater than 90% in patients whose serum HA concentration reached levels 40 g/L. Moreover, survival benefit was observed in patients even when on‐treatment serum HA did not normalize.

**Table 1:** Comparison of ANSWER and MACHT trials on long‐term albumin infusion in decompensated cirrhosis patients.

Ref	Study design	Country	Study population	Intervention	Primary endpoint	Median duration of albumin administration	Length of treatment	Sample size (intervention/control)	MELD (intervention/control)	Effects of HA
Caraceni P *et al*.^19^	Multicenter open label RCT (ANSWER trial)	Italy	Patients with moderate or large ascites requiring high dose diuretics	40 g twice per week for first 2 weeks then 40 g thereafter	Overall survival	14.5 months	18 months	431 (HA 213/SMT 218)	12 (10–15)/13 (10–16) median (IQR)	‐Increased survival in the albumin arm ‐Reduction in incidences of refractory ascites and cirrhosis complications ‐Steady and significant increase of serum HA in albumin arm (0.6–0.8 g/dL)
Sola E *et al*.^20^	Multicenter double‐blind RCT (MACHT trial)	Spain	Patients with ascites on waiting list for liver transplantation	40 g every 2 weeks plus midodrine adjusted to mean arterial pressure (no loading dose)	Incidences of cirrhosis‐related complications	63 days	12 months	196 (Midodrine and HA 99/placebo 97	17 ± 6/16 ± 6.2 (mean ± SD)	‐No difference between the 2 arms ‐Slight decrease in plasma renin activity and aldosterone in midodrine and albumin arm ‐No significant increase in serum HA in albumin arm as compared with placebo

HA, human albumin; SMT, standard medical therapy.

The pilot study PRECIOSA was an open label, nonrandomized, prospective study aimed to identify the adequate HA dose critical to increase the concentration of serum albumin to a normal range (3.4–4.7 g/L). HA was given for 12 weeks to a cohort of decompensated patients with secondary hypoalbuminemia, circulatory dysfunction, portal hypertension, and markers of systemic inflammation.^17^ Two doses were compared: 1.5 g/kg every 10 ± 2 days (maximum 100 g per patient) *versus* 1 g/kg every 2 weeks. Data were collected from 18 patients without bacterial infections and the effect on plasma cytokines was measured in bio‐banked samples from an additional 78 patients (INFECIR‐2 study). High doses of HA were associated with normalization of serum albumin, improvement of left ventricular function, and a reduction of inflammatory cytokines such as interleukin (IL)‐6, granulocyte colony‐stimulating factor (G‐CSF), IL‐1 receptor antagonist (IL‐1ra), and vascular endothelial growth factor (VEGF).

Currently, the American Association for the Study of Liver Diseases (AASLD) practice guidelines do not recommend long‐term HA treatment in patients with cirrhosis and ascites, citing cost‐effectiveness as a primary deciding factor.^13^ Importantly, the ANSWER study demonstrated a significant reduction in complications and readmissions following long‐term HA supplementation, contributing to a favorable incremental cost‐effectiveness ratio.^16^ On the contrary, when compared with SMT, a position statement from the Italian Association for the study of liver (AISF) and a recent international position statement by an academic group with special interest in HA representing contributors from all five continents recommend the long‐term HA use specifically of 40 g weekly with a target of serum albumin after 1 month reaching ≥40 g/L.^23^, ^24^

## Albumin in patients with AKI and HRS

Patients with cirrhosis and ascites can develop renal dysfunction referred to as HRS.^25^ AKI in the setting of cirrhosis (HRS‐AKI) is a common and life‐threatening complication.^26^ It is defined as functional progressive kidney failure due to severe renal vasoconstriction in the setting of splanchnic vasodilation^27^, ^28^. It is initially reversible but can lead to permanent damage associated with poor prognosis. Unlike pre‐renal AKI, which follows fluid/blood loss resulting in kidney hypoperfusion, HRS‐AKI occurs due to severe reduction of effective volume secondary to cardiocirculatory dysfunction.^29^ The traditional notion that HRS is merely a “functional” kidney injury instigated by portal hypertension has recently been challenged with novel research demonstrating the role of oxidative stress, systemic inflammation, and bile‐related tubular damage in its pathogenesis as additional “structural‐histologic” elements.^30^, ^31^

In contrast to pre‐renal AKI, HRS‐AKI does not improve following plasma volume expansion.^29^, ^32^, ^33^ Therefore, current recommendations from the International Club of Ascites (ICA) suggest HA infusion for HRS treatment at a dosage of 1 g/kg up to a maximum of 100 g daily for at least 48 h with diuretic withdrawal, followed by 20–40 g daily, ideally titrated with central venous pressure or additional parameters of blood volume, to decrease the risk of fluid overload, pulmonary edema, and respiratory failure.^32^ HA infusion may be terminated if serum albumin is >45 g/L and should be temporarily withdrawn in patients showing signs of circulatory overload, CVP >15 cm of H_2_O, and pulmonary edema. Generally, HA is the first‐choice plasma volume expander in this context, but treatment of HRS may include a combination of HA and vasoactive medications such as midodrine, octreotide, terlipressin, and norepinephrine.^34^, ^35^, ^36^ There is evidence suggesting that concomitant use of HA and vasoactive medication in particular terlipressin significantly increase the recovery of renal function in patients with HRS‐AKI. Although terlipressin improves kidney function and is the vasoconstrictor of choice in HRS management, it is associated with serious adverse events, in relation to tissue ischemia and respiratory failure (CONFIRM study).^37^, ^38^, ^39^

EASL recommends that after 2 days of HA administration (1 g/kg) for differential diagnosis, a dose of 20–40 g daily should be maintained until a complete response (i.e., serum creatinine <1.5 mg/dL) or maximal duration of 14 days,^15^ while AASLD recommends 1 g/kg on day 1, followed by 40–50 g every subsequent day.^13^ In contrast with other indications for the use of HA, these recommendations to use HA specifically for AKI are based on expert opinion rather than RCTs or prospective studies.

## Albumin in patients with bacterial infections

Spontaneous bacterial peritonitis (SBP) is the most common bacterial infection in cirrhosis. It is defined as the development of bacterial infection of ascitic fluid in the absence of any intra‐abdominal surgically treatable source of infection.^15^, ^40^, ^41^ Renal impairment is a common complication in patients with SBP and can develop in nearly a third of patients.^42^, ^43^

The benefits of HA in the context of SBP can be attributed to its capacity to inhibit inflammation and improve hemodynamic status. A study by Sort *et al*. of 126 SBP patients has shown that plasma expansion with HA at infection diagnosis improves circulatory function, markedly reduces the episodes of HRS‐AKI and hospital mortality, and increases the 90‐day probability of survival in patients with SBP. The dosage of HA was 1.5 g/kg at diagnosis and 1 g/kg on day 3.^44^ This RCT proved that HA infusion was effective even in patients with baseline serum bilirubin level ≥4 mg/dL or sCr level ≥1 mg/dL. A study by Chen *et al*. explored the effects of HA on inflammatory mediators in 30 cirrhotic patients with SBP.^45^ Patients were randomly assigned to treatment groups containing antibiotics alone (*n* = 15) or antibiotics combined with HA for the first 3 days following SBP diagnosis (*n* = 15). Antibiotics plus HA were found to significantly decrease tumor necrosis factor (TNF)‐α and IL‐6 levels in blood and ascites fluid. Another unblinded RCT compared HA use with a synthetic plasma expander to prevent the progression of renal impairment in patients with SBP. Patients were randomized to receive HA (*n* = 10) or hydroxyethyl starch (*n* = 10).^46^ Cirrhotic patients with SBP treated with HA had significant surge in arterial pressure and an inhibition of plasma renin activity, suggesting an improvement in circulatory function. Furthermore, there was major decrease in von Willebrand‐related antigen plasma levels, and simultaneous rise in the levels of serum nitrates and nitrites, suggesting an impact of HA on endothelial function. These studies demonstrate a clear physiological advantage of utilization of HA in cirrhotic patients with SBP.

LVP is not contraindicated for patients with SBP, despite the risk of post‐drain renal dysfunction. Therefore, if LVP is indicated in SBP patients, then this needs to progress with HA support.^14^ The administration of HA at a dose of 1.5 g/kg of body weight on the day of diagnosis and 1 g/kg of body weight on day 3, particularly those with baseline serum bilirubin level ≥4 mg/dL or serum creatinine level ≥1 mg/dL, reduces the likelihood of AKI and improves survival in patients with SBP.^24^, ^47^

Non‐SBP infections including urinary tract infections, pneumonia, skin infections, bacteremia, and sepsis are common in cirrhosis.^40^ However, the effect of HA on non‐SBP infection remains to be elucidated, and there is insufficient high‐level evidence to recommend the use of HA in patients with decompensation for treatment of non‐SBP infections without septic shock (Infecir‐2 study).^24^, ^48^, ^49^, ^50^ A higher incidence of pulmonary edema was reported in at least two of three RCTs.

## Albumin in patients with HE

HE is a highly prevalent neuro‐cognitive complication of cirrhosis characterized by cognitive dysfunction, and high rates of mortality and recurrence.^51^, ^52^, ^53^ An episode of HE is typically induced by a participating event such as gastrointestinal bleeding, constipation, infection, or renal failure. Hyperammonemia, inflammation, oxidative stress, and endothelial dysfunction play an essential role in the development of HE.^54^, ^55^ Even after recovery from episodes of overt HE, vast number of patients persistently continue to be cognitively impaired with minimal hepatic encephalopathy (MHE).^56^ Studies have demonstrated a higher incidence of overt HE in cirrhotic patients with hypoalbuminemia, particularly in those with a serum albumin level ≤31.6 g/L. In addition, patients with overt HE and severe hypoalbuminemia, where serum albumin was ≤22.8 g/L, were more likely to die from HE‐associated mortality.^57^

Current therapies for HE are limited in efficacy. The primary mechanism of action targets the intestinal production and absorption of ammonia with medications such as lactulose and rifaximin.^58^, ^59^ The use of HA as preventive or treatment strategy for HE is not supported for acute episodes of overt HE, and further studies are required to address this frequent complication of cirrhosis.

HA may reduce the severity of HE in hospitalized patients with overt HE, and its use is associated with a significantly higher survival than standard of care according to number of studies.^18^, ^19^, ^60^, ^61^, ^62^ A study by Simón‐Talero *et al*. assessed the efficacy of HA in a multicenter, prospective, double‐blinded, randomized controlled trial in patients with an acute episode of HE.^62^ Patients were randomized to receive HA (*n* = 26) (1.5 g/kg on day 1 and 1.0 g/kg on day 3) or isotonic saline (*n* = 30), in addition to standard medical care (laxatives, rifaximin 1200 mg per day). Results showed that there was no difference between both groups in terms of the percentage of patients without HE at day 4 (HA: 57.7% *vs* saline: 53.3%; *P* > 0.05), although significant differences in survival were found at Day 90 (HA: 69.2% *vs* saline: 40.0%; *P* = 0.02). In 2016, another RCT explored the addition of HA in 120 patients with overt HE and cirrhosis receiving lactulose. Results confirmed that combination of lactulose plus HA was more effective than lactulose alone with respect to resolution of overt HE.^60^ In addition, 10‐day mortality and hospital stay were significantly lower in the lactulose plus HA group *versus* lactulose alone (10‐day mortality18.3% *vs* 31.6%, *P* = 0.04; hospital stay 6.4 ± 3.4 *vs* 8.6 ± 4.3 days, *P* = 0.01). Furthermore, significant decrease in the levels of arterial ammonia, IL‐6, IL‐18, TNF‐α, and endotoxins was noted. A meta‐analysis including 708 patients without pre‐existing overt HE showed that HA infusion significantly reduced the incidence of overt HE (4.20% *versus* 12.70%, *P* < 0.001) and subsequent in‐hospital mortality (1.70% *versus* 5.40%, *P* = 0.008).^61^ The same study highlighted that among the 182 patients with overt HE at the time of admission or during hospitalization, HA infusion improved overt HE (84.60% *vs* 68.10%, *P* = 0.009) and reduced in‐hospital mortality (7.70% *vs* 19.80%, *P* = 0.018). A recent study in 2022 by Fagan *et al*. (HEAL study) investigated HA use in outpatients (*n* = 24) with cirrhosis and minimal HE (MHE).^63^ Patients received weekly infusions of 25% IV HA1.5 g/kg over 5 weeks. Results demonstrate that patients receiving HA had significantly higher rates of reversal and improvement of MHE as demonstrated by improvement of their cognitive testing and health‐related quality of life. It was hypothesized that these changes are likely to be secondary to HA's potential role in reduction of inflammation and endothelial dysfunction.

Despite the heterogeneity of the aforementioned studies, results of a recently published international position statement on the use of HA for cirrhosis indicates that HA at a dosage of 20–40 g daily can be considered to treat overt HE, especially in cirrhotic patients with hypoalbuminemia.^24^

## Hyponatremia

Hyponatremia in patients with cirrhosis is defined as sodium below 135 mEq/L.^64^ The most frequent type of hyponatremia is hypervolemic and hypo‐osmolar.^15^, ^65^ HA can counteract effective hypovolemia, which leads to the activation of the RAAS, and secondary water and sodium retention.

Although HA is considered a potential adjuvant treatment for hyponatremia and is included in the current international guidelines and position statements for selected patents with cirrhosis, evidence on HA use for the treatment or prevention of hyponatremia is limited. Most studies evaluating HA in hyponatremia are retrospective in nature or examine HA in the context of hyponatremia as a secondary analysis rather than a primary aim. One study included 1126 patients with cirrhosis and hyponatremia, of whom 777 received HA infusion. Results demonstrated that short‐term HA infusion improved the resolution of hyponatremia and improved serum sodium concentration (69% *vs* 61%, *P* = 0.008).^66^ Similarly, a post hoc analysis of the data from the ATTIRE trial concluded that HA administration can increase serum sodium in hyponatremic patients hospitalized with an acute decompensation but did not impact clinical outcomes.^18^ Further, a secondary analysis of the ANSWER study demonstrated that long‐term HA treatment in relatively stable outpatients with cirrhosis and ascites can improve the correction rate of hyponatremia and reduce the incidence of further episodes of hyponatremia.^16^

## Acute decompensation of cirrhosis

The ATTIRE study included 777 hospitalized cirrhotic patients with hypoalbuminemia (<30 g/L) hospitalized for acute decompensation and were assessed for short‐term repeat HA infusions *versus* SMT.^18^ The treatment arm received daily intravenous infusions of HA to increase HA level to 30 g/L throughout the trial up to 2 weeks. There were no significant differences in the probability of a composite primary endpoint event (any infection, renal dysfunction, or death during hospitalization) between treatment and control groups, despite the HA group receiving a significantly higher HA dose than the control group. The HA group also had more severe or life‐threatening serious adverse events, especially pulmonary edema, or fluid overload; however, mortality was similar between the two study arms. A protocolised aim for HA for inpatients is viewed as risky proposition. Furthermore, a combined endpoint, which includes infection, would be difficult by itself. Based on current evidence, the use of repeated infusions of HA is currently not supported for hospitalized decompensated patients or for preventing the development of further complications of the disease during hospitalization.

## Current utilization of HA in Australia

Australia is one of the few countries outside of Italy where outpatient HA infusions is accepted clinical practice in many hepatology centers. Despite this, there remains a paucity of local data on short‐ and long‐term HA use for patients living with liver cirrhosis,^67^, ^68^ with the largest Australian study evaluating long‐term use of HA to date including only 24 patients. Nevertheless, this study showed a significant reduction in portal hypertensive‐related hospital admissions and a reduced number and volume of paracentesis resulting from regular outpatient HA infusions.^67^

Long‐term HA use is not limited to indications that closely mirror the inclusion criteria of ANSWER trial. Selected patients with ascites requiring regular paracentesis, or ascites with limitations in diuretic titration (e.g., poor renal function, hyperkalemia, and hyponatremia) may be considered for long‐term HA infusions. There is a consensus that long term use of HA should be initiated early in the course of cirrhosis progression. Conversely, recent real‐world data from Italy and real‐life experience from Australia suggest that long‐term HA administration can be expanded to indications beyond the inclusion criteria of the ANSWER Study.^67^, ^69^, ^70^ This includes expansion of criteria to patients with refractory ascites prescribed additional diuretics and having more frequent LVP than the ANSWER patients (Table 2).

**Table 2:** Real‐world evidence supporting the beneficial effects of long‐term albumin use in decompensated cirrhosis.

Reference	Study design	Study population	Sample size (HA group)	Duration and dose of albumin administration	Effects of long‐term HA
Hannah *et al*.^76^	Single‐center retrospective cohort	Patients with cirrhosis and diuretic resistant ascites requiring LVP, hepatic hydrothorax, or severe peripheral edema	24 patients	40 g every 2 weeks, and one patient had 40 g every 3 weeks at least ≥1 months	‐Reduction in portal hypertensive related hospital admissions ‐Improvement in serum sodium, albumin levels, and CPS
Laleman *et al*.^79^	Multicenter retrospective cohort	Patients with cirrhosis and ascites	2355 patients	87 (10–280) g/week, followed by 37 (10–60) g/week for ≥6 months	‐Lower incidence of paracentesis ‐Lower incidence of cirrhosis related complications: refractory ascites, SPB, HRS and HE
Laleman *et al*.^80^	Multicenter retrospective cohort	Patients with decompensated cirrhosis presenting with ascites	125 patients	≥40 g per week for ≥3 months	‐Reduction in mean annual number of therapeutic paracenteses episodes by 47.8% ‐Significant reduction in refractory ascites, SBP, HRS ‐Reduction in hospital admissions and length of stay

CPS, Child‐Pugh score; HA, human albumin; HE, hepatic encephalopathy; HRS, hepatorenal syndrome; LVP, large volume paracentesis, SPB, spontaneous bacterial peritonitis.

**Figure 3:**
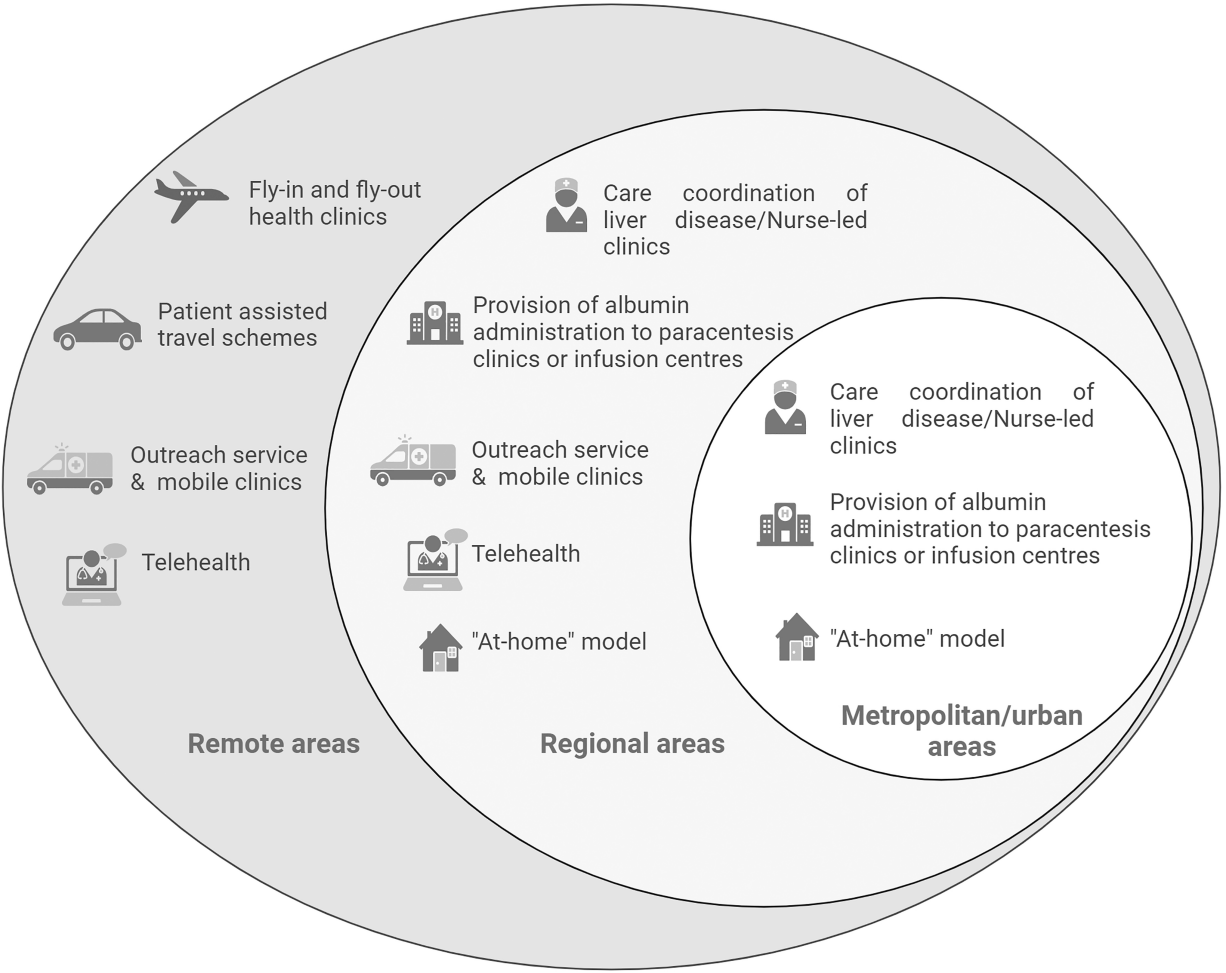
Proposed solutions to overcome logistic barriers for implementation of long‐term albumin use in clinical practice.

Contemporary healthcare models for cirrhosis care in Australia focus on the provision of centralized specialist cirrhosis care at larger district or tertiary hospitals in metropolitan areas.^71^ Remarkably, such models that involve weekly patient presentation at hospital day centers for HA infusions appears to be well tolerated and with good attendance in our Australian experience. The setting of where HA infusions are administered are center dependent, but include designated chairs in day infusion centers, paracentesis clinics, oncology day units, or other ambulatory care settings with appropriate nursing and medical supervision. Specialist hepatology nurses or nurse consultants are often engaged to oversee the booking of long‐term HA infusions as part of chronic liver disease care models, with the infusion appointments also serving as chance to engage patients in opportunistic care. However, with approximately one third of the Australian population living in regional or remote areas, such centralization poses a challenge for timely access to HA infusions. Patients in regional and remote areas often need to travel long distances for treatment, imparting a significant burden on patients and the healthcare system.^72^ Given current and predicted need, alternative healthcare delivery models have been proposed to help improve access and continuity of services for patients requiring long‐term HA infusions. Potential initiatives to overcome these barriers include outreach infusion service or outsourcing of hospital services to an “at home” model. Typically, HA infusions utilizing an “at home” model would be provided by primary care physicians or by specialist nurse consultants experienced in liver disease. Transitioning to an “at home” model of HA infusion requires transformational changes and is likely to be hindered by a number of factors such as how such services are funded, patient engagement, suitability of the home environment, and practicality of delivering or dispatching HA as it is pharmacologically classified as a blood product in Australia. A nurse‐led HA infusion service has also been proposed as another alternative to deliver HA to patients with cirrhosis (Fig. 3). Interestingly, a report from Victoria has demonstrated that a nurse‐led service has reduced the need for therapeutic paracentesis.^73^ The principles of HA use in Australia remain consistent across various etiologies of cirrhosis. Further research is warranted to investigate whether there is any difference between the use of HA and etiology of decompensated cirrhosis.

Additional challenges facing HA use in Australia are lack of suitable biomarkers to assess response to therapy and in vitro tools to uncover synergies that translate clinically to assess the treatment shortening potential and determine stopping rules. At present, it remains challenging to identify optimum HA dose and whether a one‐dose‐fits‐all regimen is adequate. Indeed, the risk of pulmonary edema and fluid overload exists in all patients.^74^ Future efforts should also concentrate on refining the target population that most benefits from long‐term HA therapy and to define regimens and individualized therapy based on disease severity and complications, while considering scientific and operational perspectives (see Box 2). Furthermore, although there are few published local data on outcomes of patients receiving long‐term HA infusion, a national cost‐effectiveness study is underway that also includes collection of data on clinical outcomes.

Box 2 **Main barriers for long‐term human albumin (HA) use for decompensated cirrhosis in Australian clinical practice**

**Table:** 

Limitations of HA use to conventional indications
2 Barriers for Access /logistics: Infusions limited to hospitals, classification of HA as a blood product
3 Absence of well‐defined target population that benefits the most from long‐term HA therapy
4 Horizontal inequity to access HA for patients of regional and remote areas
5 Lack of optimum HA dosage
6 Potential risk of adverse effects (pulmonary edema, fluid overload, allergic reactions, etc.)
7 Lack of biomarkers of response
8 Absence of stopping rules
9 Absence of definite regimens based on disease severity and complications
10 Quality of commercially available HA
11 Pharmacoeconomics and cost‐effectiveness
12 Paucity of real‐life data for research

## Quality of the commercially available HA

In Australia, HA is supplied by a single provider (CSL Behring). Formulations are manufactured from human plasma collected from Australian Red Cross Lifeblood and prepared using predominantly chromatographic techniques. While devices have been developed to eliminate industrial stabilizers and contaminants, current HA formulations in Australia still contain industrial stabilizers such as caprylate (octanoate) and acetyltryptophanate. These compounds have shown to accumulate in patients with hepatic dysfunction and to induce vasodilatation that may influence the capacity of HA to restore or maintain renal function.^3^, ^75^ Apart from that, available formulations may contain no more than 50% HA in functionally active/reduced form.^76^

## Pharmaco‐economics

The cost of HA infusions remains a significant challenge for long‐term HA use. In Australia, HA is currently in a cost share arrangement between the federal government and the state government. In some states, the cost is passed on to the hospital. Under the national blood authority's guidance, the listing price for 20 g/100 mL of HA is AUD $71.97 (Table 3).

**Table 3:** The list price of albumin in Australia (Source: NBA website https://www.blood.gov.au/national‐product‐price‐list, July 2023)

Product type	Name	Presentation	Supplier	Price^†^
Albumin	Albumex	20% 10 mL	CSL Behring	$18.25
		20% 100 mL		$71.97
		4% 50 mL		$18.25
		4% 500 mL		$71.97
Albumin	ALBUREX 20 AU	10 g/50 mL		$91.26
		20 g/100 mL		$71.97

^†^ National product price list on first July 2023.

Major challenges relating to the access of HA exist. A lack of funding and resources for hospital ambulatory care services is a significant factor, which affects access to HA infusions and largely limits its use to hospitals. A cost–benefit study assessing HA use in Australia is warranted.

Despite the perceived financial burden of incorporating long‐term HA into SMT, results from the ANSWER trial suggest that the associated costs could be balanced out by decreases in complications, hospital admissions, and the need for acute HA.^16^ Economic evaluations in Brazil and Mexico suggest that long‐term HA use could be cost‐saving, particularly with regard to healthcare resource use.^77^, ^78^ Similarly, a study across three countries (Germany, Italy, and Spain) found HA to be both more effective and less costly than saline, gelatin, or no fluid when treating LVP.^79^ The same study has shown in both Germany and Italy that the combination of HA and antibiotics was also less costly than antibiotics alone for SBP. Furthermore, albumin plus a vasoconstrictor was both more effective and less costly than using a vasoconstrictor alone for treating HRS. In contrary, a study from Thailand demonstrated that terlipressin/noradrenaline and albumin treatments for HRS was not cost‐effective compared with SMT.^80^ Remarkably, a report from Indonesia has shown that HA may be a cost‐effective treatment for SBP, HRS, and LVP in resource‐limited healthcare settings.^81^

## Conclusion

For decades, HA has been administered to patients to provide adequate oncotic pressure and to treat hypovolemia. Currently, improved insights regarding HA's biochemical properties and physiologic functions have expanded its application beyond classical indications. In the complex network of pathophysiological pathways that underlie decompensated cirrhosis, HA represents a promising new multitargeting player in the management of cirrhosis. However, from a clinical standpoint, the use of long‐term HA for novel indications remains controversial and is primarily due to paucity in/heterogeneity of RCT designs and lack of high‐level evidence. In addition to this controversy is the lack of optimal infusion strategy and dosage, pharmaco‐economics, potential adverse reactions, and lack of proper criteria for identification of patients who would benefit mostly from HA infusions. Meanwhile, the overall Australian experience with HA infusions has been largely successful, with Australia being among the very few countries worldwide to early adopt outpatient HA infusion as well as HA use for long term.
